# Levcromakalim provokes an acute rapid-onset migraine-like phenotype without inducing cortical spreading depolarization

**DOI:** 10.1186/s10194-023-01627-9

**Published:** 2023-07-24

**Authors:** Berkay Alpay, Bariscan Cimen, Elif Akaydin, Hayrunnisa Bolay, Yildirim Sara

**Affiliations:** 1 Department of Medical Pharmacology, Faculty of Medicine, Hacettepe University, Sihhiye Ankara, Türkiye; 2 Neuroscience and Neurotechnology Excellence Joint Application and Research Center (NÖROM), Ankara, Türkiye; 3 Department of Neurology and Algology, Faculty of Medicine, Gazi University, Besevler Ankara, Türkiye

**Keywords:** Levcromakalim, Migraine, Aura, Cortical spreading depolarization, Rat model, c-fos, Allodynia

## Abstract

**Background:**

Migraine headache attacks and accompanying sensory augmentation can be induced by several agents including levcromakalim (LVC), that is also capable of provoking aura-like symptoms in migraineurs. We investigated whether single LVC injection causes acute migraine-like phenotype in rats and induces/modulates cortical spreading depolarization (CSD), a rodent model of migraine aura.

**Methods:**

Wistar rats were administered LVC (1 mg/kg, i.p.) and compared to control (CTRL, vehicle, i.p.) and nitroglycerin (NTG, 10 mg/kg, i.p.) groups. Von Frey filaments were used to examine the periorbital and hind paw mechanical allodynia. Dark–light box (DLB), elevated plus maze (EPM), and open field arena (OFA) were used to evaluate light sensitivity and anxiety-related behaviors. The effects of LVC on CSD parameters, somatosensory evoked potentials, and baseline dural EEG (electroencephalography) were investigated. Possible CSD-induced c-fos expression was studied with Western Blot. Blood–brain barrier integrity in cortex was examined with Evans blue assay.

**Results:**

LVC and NTG administration robustly reduced periorbital mechanical thresholds in rats and induced anxiety-like behaviors and photophobia within 30 and 120 min, respectively. LVC induced migraine-like phenotype recovered in 2 h while NTG group did not fully recover before 4 h. Both LVC and NTG did not provoke DC (direct current) shift, EEG alterations or cortical c-fos expression characteristic to CSD. LVC did not induce de novo CSD and affect KCl (potassium chloride)-induced CSD parameters except for an increase in propagation failure. However, NTG significantly increased both CSD susceptibility and propagation failure. Somatosensory evoked potential (SSEP) configurations were not altered in both LVC and NTG groups, but SSEP latencies were prolonged after CSD. Acute LVC or NTG injection did not increase cortical BBB permeability.

**Conclusions:**

Single LVC administration induced the fastest manifestation and recovery of acute migraine-like phenotype which was not mediated by CSD waves in the cerebral cortex. We suppose LVC triggered rapid-onset migraine-like symptoms are probably related to functional alterations in the trigeminal nociceptive system and K^+^ channel opening properties of LVC. Understanding the neurobiological mechanisms of this nociceptive window, may provide a novel target in migraine treatment.

## Introduction

Migraine is a primary headache disorder hampering the quality of life of millions worldwide [1]. Migraine attacks are characterized by unilateral pulsating frontotemporal moderate to severe pain lasting 4–72 h, and associated photophobia, phonophobia, nausea and vomiting. Approximately one fourth of migraineurs may experience aura symptoms mainly manifested in the visual sensory domain, preceding or accompanying the headache phase [2].

Several pharmacological agents, such as nitroglycerin (NTG), calcitonin G-related peptide (CGRP), cilostazol, are commonly used to provoke migraine headache attacks in migraine patients and seldomly, NTG was shown to trigger migraine aura [3–6]. However, in a clinical trial of Al-Karagholi et al., levcromakalim (LVC), an ATP-gated potassium channel opener, robustly triggered migraine aura-like phenomena in migraineurs with aura [7]. Therefore, LVC is unique in terms of its strong aura-inducing properties when compared to other migraine-inducing substances.

Preclinical rodent models of migraine have been commonly used to develop new therapies to tackle migraine burden and to explain pathophysiological mechanisms behind the disorder. In that regard, a well-characterized and clinically relevant migraine model is NTG-induced migraine model. NTG was shown to induce headache-like phenotype in rodents, as evidenced by mechanical threshold reduction in the periorbital region as a correlate of cephalic allodynia and activation of trigeminal nucleus caudalis (TNC) [8, 9]. TNC activation can be also detected by electrophysiological recordings as enhanced firing of the neurons [10, 11]. This increased firing manifests phenotypically as mechanical allodynia [8, 12, 13]. Besides, intraperitoneal NTG injection can induce anxiety-like behaviors and light aversion in rats, imitating concurrent anxious/depressive behavior and photophobia seen in migraineurs [13]. It is currently unknown whether single LVC injection leads to an acute migraine-like phenotype in rats, including mechanical allodynia, anxiety-like behavior, and photophobia.

Canonically, a strong depolarization wave, called cortical spreading depolarization (CSD), is thought to underlie the migraine aura phenomenon. Thus, CSD is proposed to be the neurobiological correlate of migraine aura in rodent models as well as humans [14]. NTG has been reported to induce migraine headache but not aura in migraine patients. In addition, NTG does not induce CSD or modify CSD parameters except for propagation failure [15]. It is a vital question to be established whether LVC can initiate CSD in rats and have facilitatory effects on a propagating CSD wave.

Studies have revealed the presence of a dysfunctional sensory gating in migraineurs [16]. Sensory gating dysfunction is believed to underlie the sensory augmentation in migraine patients [17]. The prior researchers have used somatosensory evoked potentials (SSEP) to investigate these deficits [18–21]. SSEPs can be quantified by their amplitudes and latencies [22, 23]. It is unclear how these SSEP parameters are altered in CSD-, NTG- or LVC-induced migraine models.

In recent years, neuroinflammation has emerged as a crucial aspect of migraine pathophysiology [24]. NTG-induced chronic migraine model has been revealed to involve neuroinflammatory processes and consequent loss of BBB integrity in TNC [25]. However, it is yet to be discovered if NTG and LVC injection induce BBB disruption in the cortex, where CSD could also contribute to this impairment.

The primary aim of this study is to investigate whether acute ip LVC administration can induce CSD and headache-like phenotype in rats. We also seek to explore if chemically induced CSD parameters are altered by LVC. Another goal of our study is to assess possible sensory processing deficits and general cortical network activity changes after a single LVC injection.

## Materials and methods

### Animals

Animal studies are reported in compliance with the ARRIVE guidelines [26]. Male Wistar Albino rats (Kobay, Türkiye) of 12 weeks of age, weighing 250–300 g at the time of arrival, were housed in groups of three in polycarbonate cages with wood shaving bedding on a 12/12 h light/dark schedule (lights on at 07:00 h) in a climate-controlled room. Rats were given at least 1 week for acclimatization prior to any procedure. All experiments were conducted between 09.00 – 17.00. All procedures were approved by the Hacettepe University Animal Experimentation Ethics Board (approval no: 2022/05–11).

### Experimental drugs and animal models of acute migraine

LVC (sc-361230A, Santa Cruz Biotechnology, Dallas, TX, USA) was dissolved in pure ethanol (1 mg LVC in 0,5 ml ethanol) and diluted in isotonic saline so that final solution contained 0,1 mg LVC per 1 ml of 5% ethanol in saline (v/v). NTG was supplied as an ethanol-based solution and diluted in isotonic saline so that final solution contained 1 mg NTG per 1 ml of 10% ethanol in saline (v/v). Both drugs were delivered through intraperitoneal route in all experiments. NTG-induced acute migraine model with a dose of 10 mg/kg is already extensively validated by many studies [8, 27–29], therefore, we adopted the same dose for our study. For LVC-induced acute migraine model, we administered 1 mg/kg LVC, a dose used in an LVC-induced chronic migraine model [30]. Ethanol in saline solutions were used as vehicles for the control (CTRL) groups.

### Experimental design

3–4-month-old rats were randomly allocated to 3 different groups: control (CTRL), nitroglycerin (NTG), and Levcromakalim (LVC). As for vehicle (VEH), both dilutions of ethanol (5% and 10%) in saline solutions were preliminarily tested in all experimental procedures and no statistical significance was found concerning the outputs of our experiments. Hence, the data of vehicle-injected rats were pooled under the same control (CTRL) group.

**Fig. 1:**
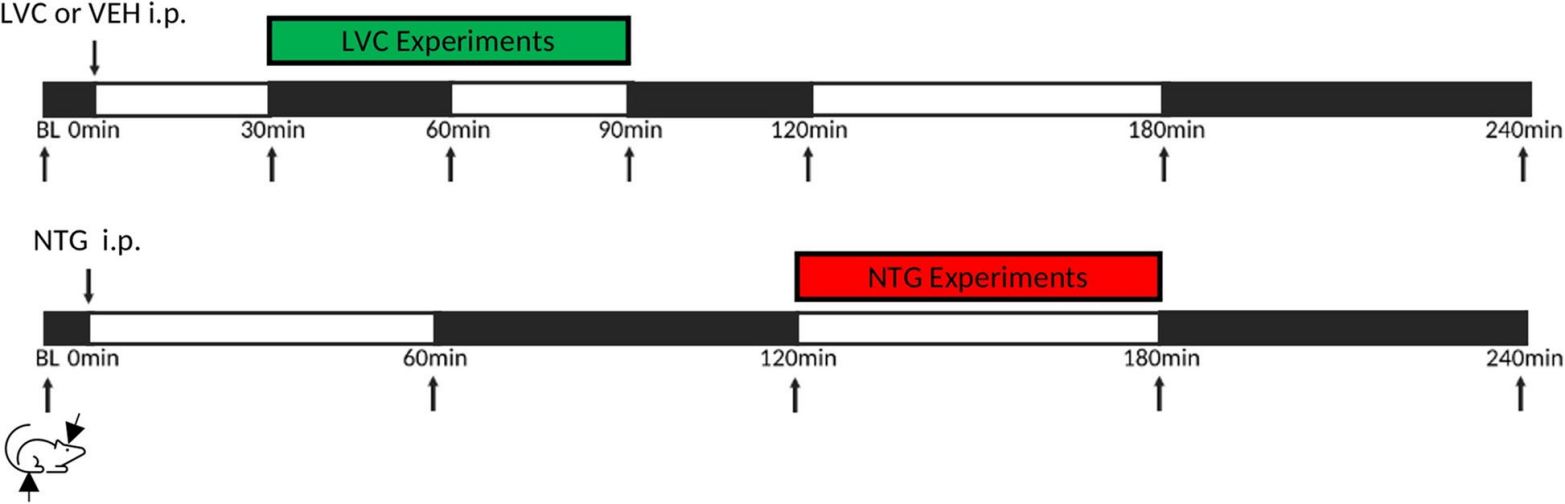
Experimental Design. Mechanical thresholds of CTRL (5% or 10% ethanol in saline) and LVC (1 mg/kg) groups were tested at baseline (BL), 30^th^, 60^th^, 90^th^, 120^th^, 180^th^ and 240^th^ min time points. NTG (10 mg/kg) group was tested at baseline, 60^th^, 120^th^, 180^th^ and 240.^th^ min. Timeframes with lowest thresholds were accepted as “headache phase” and all other experimental procedures were coincided to that timeframe (30-90 min for LVC and 120-180 min for NTG)

As a preclinical correlate of a migraine attack, we measured periorbital and hindlimb mechanical allodynia for nociceptive sensitization. Then, another set of rats were injected with vehicle, NTG or LVC and assessed for anxiety-like behavior, light aversion, and locomotion within the time frame where the lowest mechanical threshold has been detected. Vehicle-injected rats were tested at the relevant time frames of their corresponding intervention groups (10% ethanol in saline-injected rats were tested between the second and third hours of injection and 5% ethanol in saline-injected rats were tested between the 30^th^ minute and 90^th^ minute after the injection.). For in vivo electrophysiology experiments, rats were given vehicle, NTG or LVC injections and CSD parameters were evaluated at the relevant time frames (Fig. 1). Western blot experiments were performed with vehicle-injected, CSD-induced, and LVC-injected rats 3 h later following the intervention.

### Determination of sample size

Sample size for each group was calculated using G*power and resource equation. From preliminary experiments, we have determined an effect size of 0,7. Type I error (α error) probability and type II error (β error) was set at 0,05 and 0,8 respectively. These values yielded a total sample size of 24, 8 per experimental group. According to resource equation, total animals for a given experiment set – group number should be between 10 and 20. This calculation yielded 4–7 animals for each experimental group. For evans blue assay and western blot experiments, 3 rats per group were used. All sample sizes are indicated in the figure legends. A total of 88 animals was used during this study.

### Behavioral tests

#### Mechanical allodynia

Periorbital and hindlimb mechanical allodynia were evaluated with von Frey filaments with “up-and-down” method [31]. Briefly, von Frey monofilaments, starting from 2.0 g, were applied to the mid-rostral portion of both eyes for periorbital mechanical threshold testing. Aversive head withdrawals, headshakes, vocalizations, ipsilateral head scratches and whole-body retractions were accepted as positive responses. For hind paw threshold testing, rats were placed into the hind paw testing chamber. Testing started after the exploratory behavior had ended. Von Frey monofilaments were applied onto the mid-plantar part of the hind paw. Paw withdrawal, paw licking, and vocalizations were accepted as positive responses. Calculation of 50% withdrawal thresholds was done using the free online calculator at https://bioapps.shinyapps.io/von_frey_app/.

#### Anxiety-like behavior

For the evaluation of the anxiety-like behavior associated with migraine headache, elevated plus maze is used [32]. Rats were placed at the center of the plus shape maze which is constructed of four perpendicular arms (40 cm × 15 cm). Two of the arms were open and two of them were surrounded by walls (45 cm). The time spent in the open arms were analyzed for 5 min.

#### Light-aversive behavior

Aversion to light was tested with an apparatus consisting of two adjoined compartments, one being brightly illuminated (3000 lx) and the other being nearly completely dark [33, 34]. Rats were free to explore both chambers during the test. Time spent in the light compartment and number of dark-to-light transitions were measured for 15 min.

#### Open field arena test

Light-independent anxiety was evaluated with open field arena maze [35]. The maze was a transparent open glass cube (45 cm × 45 cm × 45 cm). Rats were placed at the center of the maze and allowed to move freely. The cumulative time spent in the center zone and number of transitions to center zone were recorded for 30 min. Time spent in the center zone was also considered as a measure of light-independent anxiety-like behavior [36].

### In vivo electrophysiology

Rats were anesthetized with an intraperitoneal injection of 1,2 g/kg urethane. Anesthesia depth was confirmed by the absence of toe withdrawal reflex and cornea reflex. Rats were then transferred to the stereotaxic frame and their body temperature was kept constant with a thermoregulatory blanket. A midline incision was made to expose bregma and lambda. 2 burr holes were trepanated to access right primary somatosensory cortex (AP 0.0; ML ± 3.8) and ipsilateral primary visual cortex (AP -6.0; ML -3.8). Dura was specifically left intact to avoid any pinprick induced CSDs. A third burr hole was used to chemically induce CSD. For the ground electrode a gold-plated needle electrode was inserted at the neck of the animal. AC and DC recordings over the dura mater were obtained with Ag/AgCl glass microelectrodes containing hypertonic saline. Left median nerve was stimulated every 10 s (1 mA stimulus current; 0,2 ms pulse duration) and somatosensory evoked potentials (SSEP) were recorded from right somatosensory cortex. Dural EEG signals were recorded and amplified with a head stage (Batiray, YSED, Türkiye) and an amplifier (Kaldiray EX-2C, YSED) and digitized by a data acquisition system (PowerLab 8/SP, ADInstruments, Australia). Data recordings and analysis were done by using LabChart software (AD Instruments, Australia). After 20 min of baseline recording, CSD threshold was calculated chemically by placing a cotton ball soaked in ascending concentrations of KCl (25 mM, 50 mM, 75 mM, 125 mM, 150 mM, 175 mM, 250 mM, 500 mM) with 5-min intervals [37]. Then, to induce serial CSDs, a cotton ball soaked in 1 M KCl was placed over the CSD burr hole and kept moist by impregnation with 1 M KCl every 15 min. These CSDs were evaluated in terms of amplitude, duration, frequency, and propagation failure. Evoked potentials were quantified based on their amplitudes and their latency-to-peaks. Power spectra of the baseline EEG were also computed.

### Western blot

Animals were given urethane anesthesia (1,4 g/kg) and parietal cortices were removed. Collected tissues were homogenized with RIPA lysis buffer (sc-24948A, Santa Cruz Biotechnology, Dallas, TX, USA) as indicated by the manufacturer and lysates were stored in -80 °C until further analysis. Protein concentrations were measured with BCA Colorimetric Assay Kit (E-BC-K318-M, Elabscience, USA).

Cortical protein samples (50 μg/10 μL) were loaded onto a 10% acrylamide gel (TGX FastCast Acrylamide Solution, Bio-Rad, USA), run at 90 V for 90 min, and transferred to PDVF at 1.3 mA for 7 min via Trans-Blot Turbo Transfer System (Bio-Rad, USA). Thereafter, membranes were blocked with 5% non-fat dry milk solution for 2 h at room temperature (Bio-Rad, USA). Membranes were incubated with primary antibodies (c-fos: ABE457, Millipore, USA at 1:2000 dilution; β-actin: Bioss, USA at 1:1000 dilution) overnight at 4 °C. After 3 rounds of TBS-T wash, the membranes were incubated with secondary antibody (AP307P, Millipore, USA) at 1:2000 dilution for 1 h at room temperature. The membranes were treated with Western Chemiluminescent HRP Substrate (Immobilon, #WBKLS, Merck) for 5 min in the dark. Membranes were then visualized with Syngene G:BOX Chemi XRQ and analyzed with ImageJ (NIH, USA). β-actin levels were used to normalize loaded protein. The relative change in protein levels was demonstrated as normalized ratio (c-fos/β-actin).

### Evans blue assay

Evans blue (EB) assay was performed as described previously [38]. Briefly, EB was prepared as %2 (w/v) in saline. Animals were anesthetized with urethane (1,4 g/kg) and injected with EB (3 ml/kg). All injections were timed so that animals were exposed to EB dye for at least 1 h when their mechanical thresholds were found to be lowest. During the procedure, their body temperature was kept constant with a thermoregulatory blanket. Subsequently, animals were transcardially perfused with saline. Frontoparietal and occipital cortices were collected. Wet weights of the brains were measured and 1 mL %50 (w/v in saline) trichloroacetic acid (TCA) was added per 1 g of tissue. Specimens were then homogenized and centrifuged for 20 min at 10,000 g. Supernatants are collected and diluted with ethanol four-fold. Standards were also prepared as EB dye in ethanol with various concentrations. Absorbances were measured at 620 nm with spectrophotometer (MultiScanGO, Thermo Fisher Scientific, USA).

### Statistical analyses

Data are presented as mean ± SEM. Statistical analyses were performed by GraphPad Prism (GraphPad Software Inc., CA, USA). For parametric data, student's *t*‐test, or ANOVA (one‐way or two‐way) followed by the appropriate post hoc test was used when there were two or three groups to compare, respectively. For non-parametric data, Mann Whitney U, Kruskal–Wallis and Friedmann tests were used. Post hoc tests were not performed when *F* values of ANOVA were not significant. *P* < 0.05 was considered as statistically significant.

## Results

### LVC and NTG induce hind paw and periorbital mechanical allodynia

To exclude possible threshold alterations by vehicle, we first studied the effects of ip %10 EtOH in saline (10 ml/kg) injection. We did not find any changes with this amount of ethanol treatment at any point of the time course in both periorbital and hind paw measurements.

**Fig. 2:**
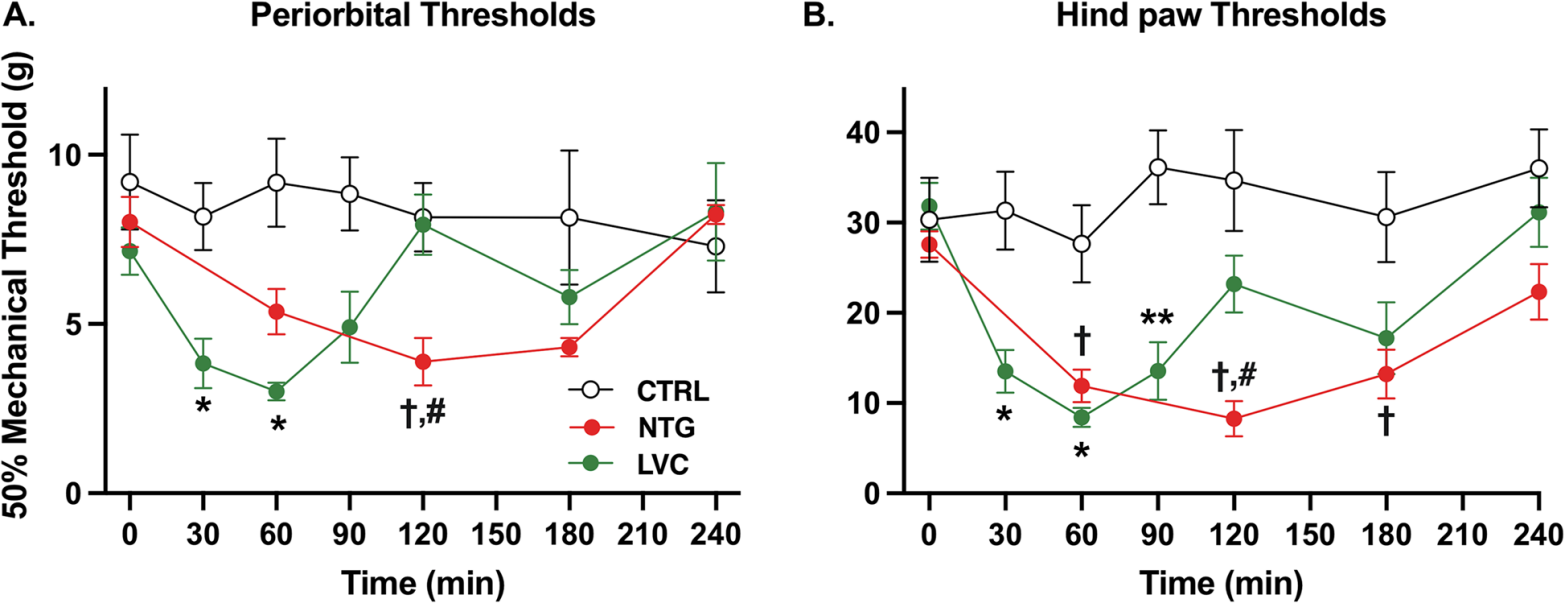
Acute LVC induced fast and brief periorbital and hind paw mechanical hyperalgesia. Both LVC and NTG injection significantly reduced mechanical thresholds in both periorbital (**A**) and hind paw plantar areas (**B**) [F(2,17) = 5,464; *p* < 0,05;]. LVC administration yielded a rapid reduction at 30 min and this hypersensitivity peaked at 60 min. LVC-induced hypersensitivity ended at 120 min. In comparison, NTG decreased thresholds in 120 min and resulted in longer term hypersensitivity that did not recover until 240 min. Data were shown as mean ± SEM. Statistical analysis was performed with two-way ANOVA and *posthoc* Tukey’s test (*p < 0,05 LVC vs CTRL; ** p < *0,01 LVC vs. CTRL; † p* < *0,05 NTG vs CTRL; # p* < *0,05 LVC vs NTG)* (*n* = 6–8/group)

Firstly, we compared the acute LVC- and NTG-induced periorbital mechanical threshold reductions over time [F(2,17) = 5,464; *p* < 0,05; Fig. 2A]. Following ip LVC injection, we observed significant reduction at 30 min, and thresholds dropped to minimum (3,00 ± 0,26) at around 60 min as compared to the respective CTRL values (*p* < 0,05) and recovered after about 120 min. NTG group exhibited reduced periorbital thresholds at the 2^nd^ hour (*p* < 0,05) and returned to baseline at 240 min.

Similarly, LVC and NTG treatment yielded a temporary reduction in hind paw mechanical thresholds [F(2,21) = 10,43; *p* < 0,001, Fig. 2B]. After LVC injection, we observed a significant decrease in thresholds at 30 and 60 min. At 60 min, the lowest hind paw thresholds (8.41 ± 1.04 g) were detected in comparison to the corresponding control values (*p* < 0,05). The thresholds recovered after approximately 120 min, consistent with the periorbital LVC thresholds. NTG injection also reduced the hind paw mechanical thresholds at the 60, 120 and 180 min time points, [*p* < 0,05 for all three timepoints]. Maximal recovery for NTG group was attained at 240 min where no statistical significance was found between NTG and CTRL group (*p* > 0,05) in line with periorbital NTG threshold values.

Our results demonstrate that LVC and NTG successfully provoke periorbital and hind paw mechanical allodynia.

### LVC and NTG induce pain-related behaviors

To further investigate whether LVC induces headache-like phenotype, we also evaluated cephalalgia-related behaviors. Since our behavioral testing battery lasted approximately 1 h for each animal, we chose the minimum points of the periorbital thresholds for the behavioral experiments. For NTG group, we initiated our tests at the 2^nd^ hour of the NTG injection. For LVC group, the experiments were timed to begin at the 30^th^ minute after the injection.

**Fig. 3:**
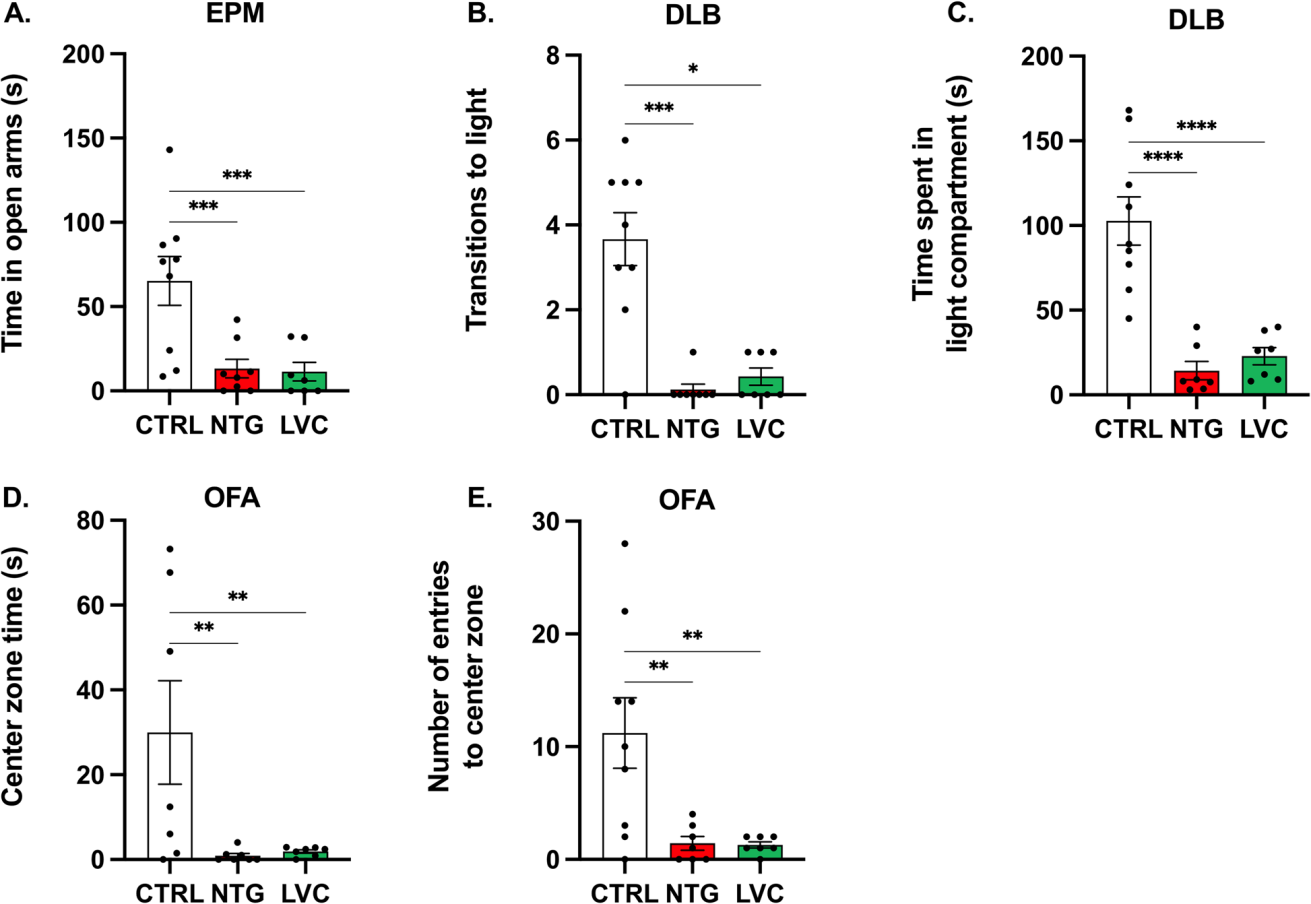
Acute LVC and NTG induced migraine-associated signs such as anxiety-like behavior and photophobia. **A** Acute LVC and NTG administered rat spent less time in the open arms of the elevated plus maze. **B** These rats were also exhibited lower number of transitions from dark to light compartments and **C**. Spent less time in the light compartment during the experiments of dark–light box. **D** After LVC or NTG injection animals spent less time in the center zone and **E** displayed fewer number of entries to this zone in open field arena test (OFA). Data are shown as mean ± SEM. Statistical analysis was performed with one-way ANOVA and *posthoc* Tukey’s test or Kruskal Wallis test and *posthoc* Dunn’s multiple comparisons test (* < 0,05; ** < 0,01; *** < 0,001; **** < *0,0001)* (*n* = 7–8/group)

In the EPM test, rats in the NTG and LVC groups spent significantly less time in the open arms of the platform indicating the presence of anxiety-like behavior [F(2,12.21) = 10,51; *p* < 0,01; Fig. 3A]. In the dark–light box (DLB) test, number of entries from dark to light compartment were significantly decreased after LVC or NTG injection (Kruskal–Wallis statistics 14,27; *p* < 0,0001; Fig. 3B). Similarly, animals spent a shorter time in the light compartment when injected with either LVC or NTG (Kruskal–Wallis statistics 16,53; *p* < 0,0001; Fig. 3C). Lastly, in the open field arena (OFA) test, we assessed the anxiety-like behaviors without interference of light. In both LVC and NTG groups, rats displayed anxiety-like behavior as became evident from the reduced time spent in the center zone [F(6,40) = 3.85; *p* < 0,05; Fig. 3D] and lower number of entries to the center zone (F (2, 20) = 7.378, *p* < 0,01; Fig. 3E). It can be inferred that LVC and NTG administration causes anxiety-like behavior and photophobia.

### LVC does not affect KCl-induced CSD parameters except for propagation failure whereas NTG lowers KCl-induced CSD threshold

**Fig. 4:**
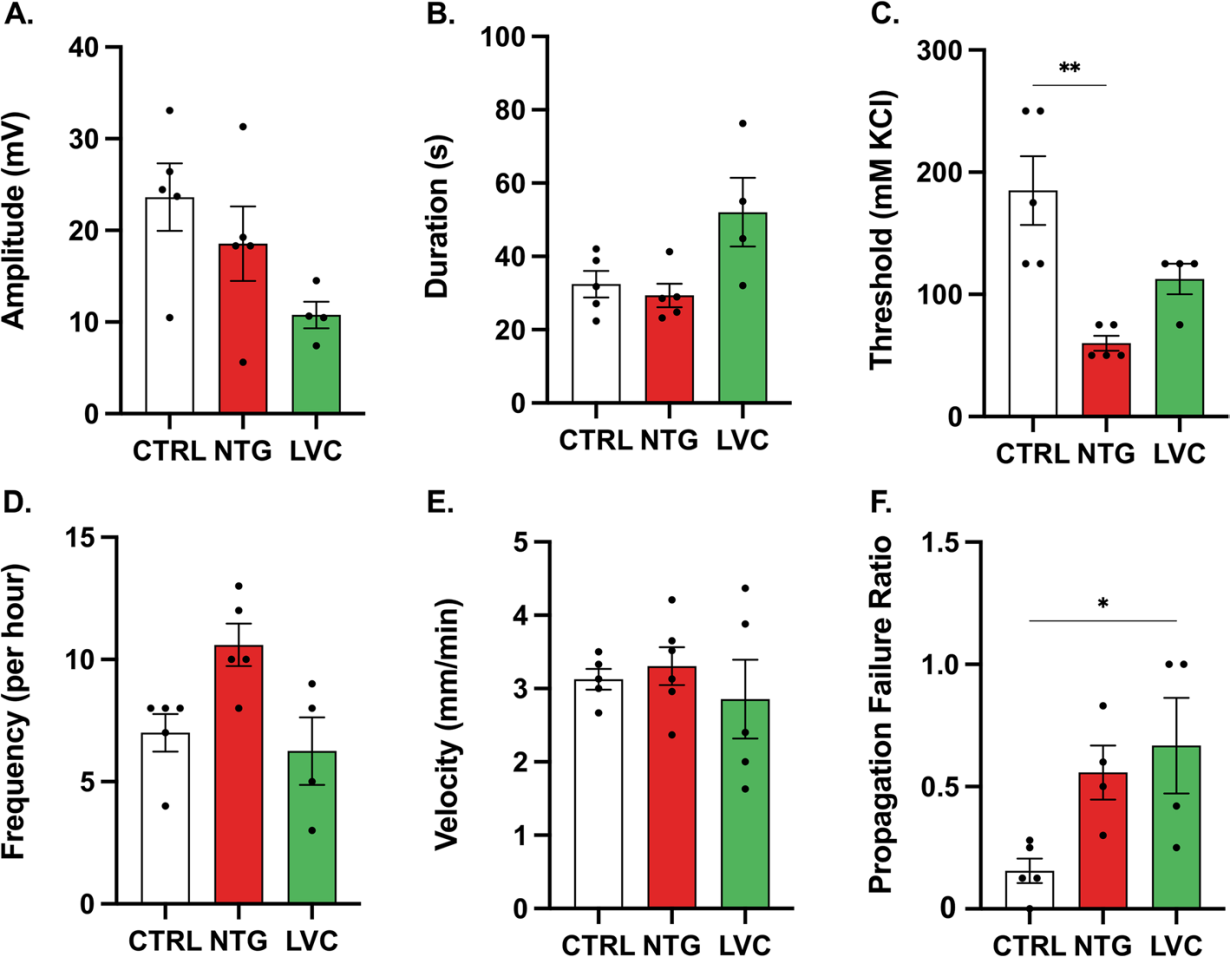
LVC and NTG both decreased propagation of CSD while NTG also increased CSD susceptibility. LVC or NTG administration did not affect CSD **A** amplitudes, **B** durations, **D** frequency and **E** velocity.** C** LVC did not change CSD thresholds, whereas NTG-injected rats had lower thresholds. **F** LVC increased propagation failure as compared to CTRL group. Data were shown as mean ± SEM. Statistical analysis was performed with one-way ANOVA and *posthoc* Tukey’s test or Kruskal Wallis test and *posthoc* Dunn’s multiple comparisons test (* < 0,05; ** < *0,01)* (*n* = 4–5/group)

To evaluate possible facilitatory effect of LVC on CSD, we have examined CSD parameters, such as threshold, amplitude, duration, frequency, and propagation failure. LVC did not affect CSD threshold, amplitude, frequency, and duration (*p* > 0,05 for all; Fig. 4A-D). LVC group showed an increased propagation failure rate (*p* < 0,05; Fig. 4F). None of the agents administered had no influence on the CSD velocities (Fig. 4E). Likewise, NTG did not change any of the aforementioned CSD parameters except for the threshold (Kruskal–Wallis statistics 10,35; *p* < 0,05) (Fig. 4A-F).

### LVC and NTG do not change peak amplitudes and latencies of SSEPs

**Fig. 5:**
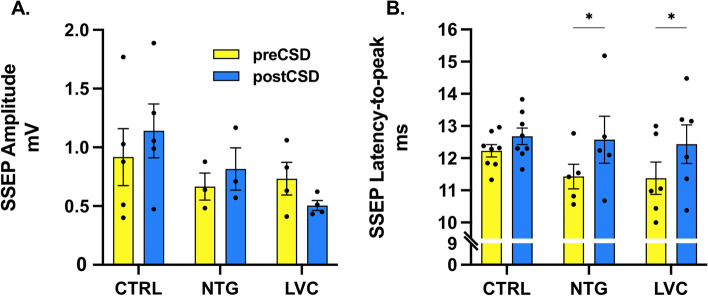
LVC or NTG administration did not change maximal SSEP amplitudes and latencies-to-peak. Overall, LVC and NTG did not change **A** SSEP amplitudes and **B** latencies. Following SD, LVC and NTG-treated groups displayed prolonged SSEP latencies. Data are shown as mean ± SEM. Statistical analysis was performed with two-way ANOVA and *posthoc* Tukey’s test (* < *0,05)* (*n* = 4–8/group)

To clarify if LVC alters the signal transduction from periphery to cortex and somatosensory cortical processing, we have utilized somatosensory evoked potentials. Neither NTG nor LVC injection changed amplitudes before or after CSD [F (2, 9) = 0.4238;*p* > 0,05 and F (2, 9) = 3.189; *p* > 0,05 respectively] (Fig. 5A). LVC- and NTG-injected rats had similar latencies to CTRL group before and after CSD [F (2, 16) = 0,58; *p* > 0,05]. When compared within each treatment group, latencies were significantly longer in NTG and LVC group following CSD [F (1, 16) = 22,27; *p* < 0,001] (Fig. 5B). SSEP configurations were similar before and after LVC injection in the LVC-treated group (data not shown).

### LVC-injected rats exhibit altered power spectrum density profile after CSD

**Fig. 6:**
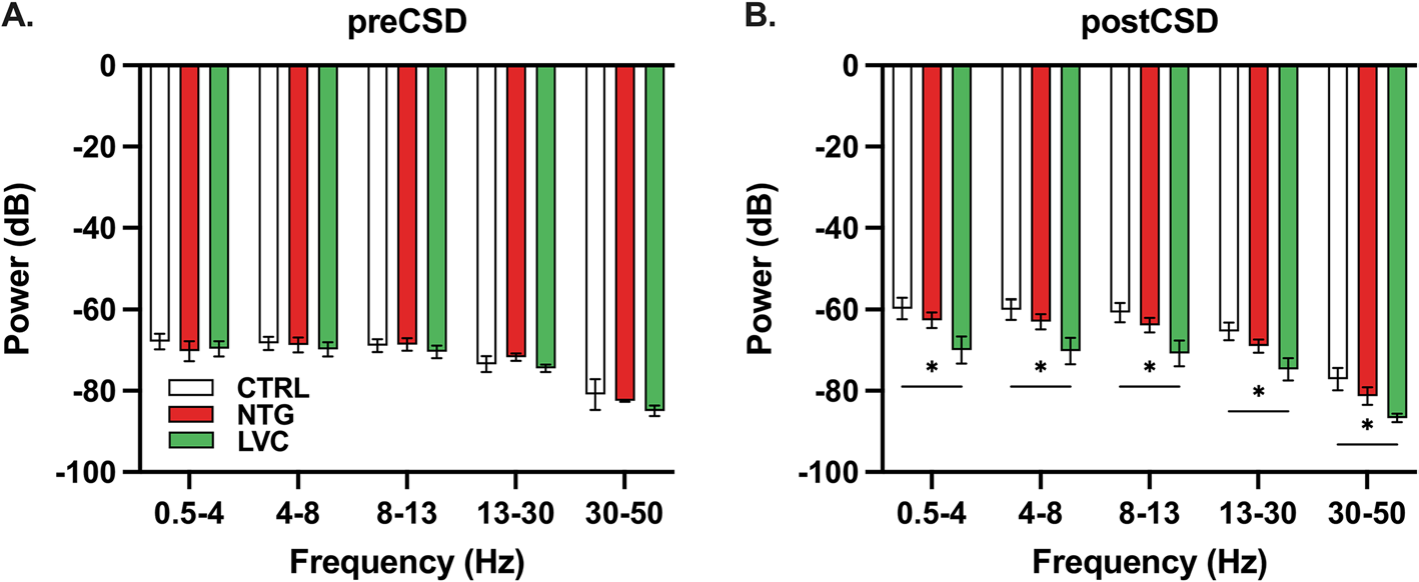
LVC-injected rats had distinct power spectrum density profiles after CSD. **A** Power spectrum density profiles were similar between different treatment groups before CSD. **B** LVC group was found to have increased activity within delta, theta, alpha, beta, and gamma ranges following SD. Data are shown as mean ± SEM. Statistical analysis was performed with two-way ANOVA and posthoc Tukey’s test (* < 0,05) (*n* = 4-6/group)

To assess general network activity, we have analyzed baseline EEG recordings for their power spectra. Before CSD, no difference was detected between different treatment groups (F(2, 88) = 2,914; *p* > 0,05) (Fig. 6A). Following an CSD wave, LVC group exhibited a weaker activity within the delta, theta, alpha, beta, and gamma range as compared to CTRL group (Fig. 6B).

### LVC does not induce c-fos expression in rat cortex

**Fig. 7:**
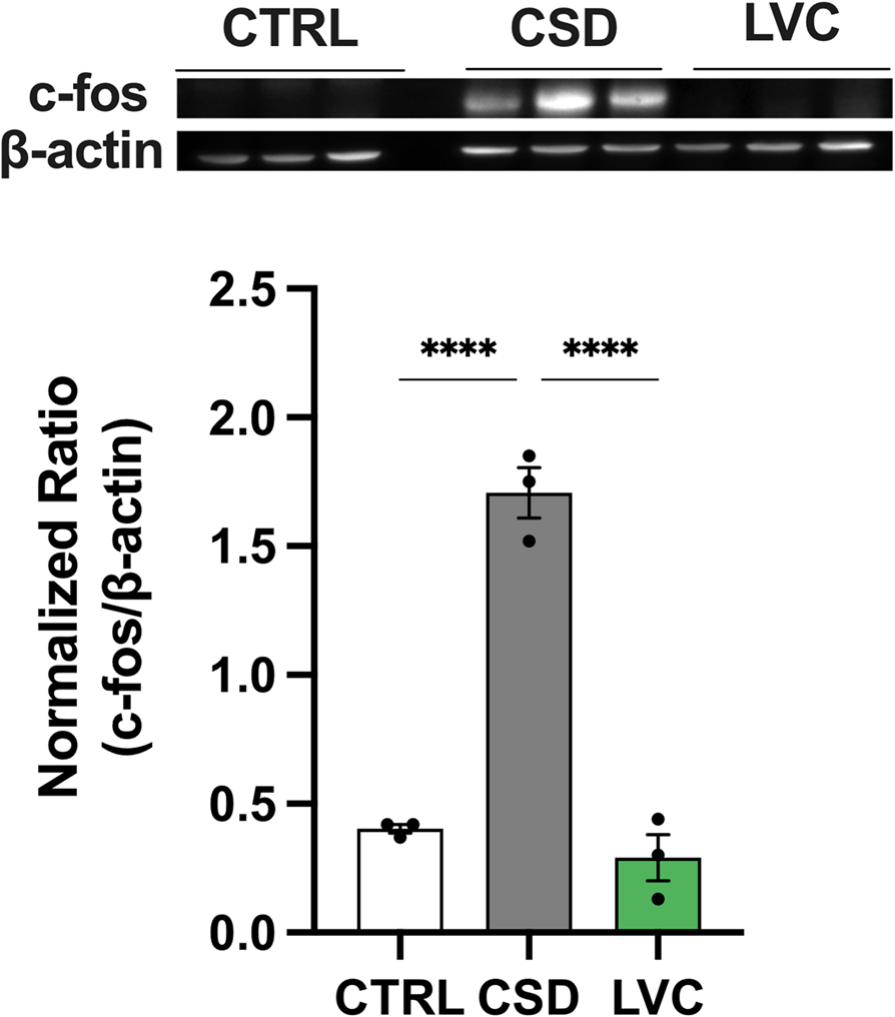
LVC did not increase c-fos expression in cortex. CSD led to increased c-fos in cortex (*p* < 0,05). LVC did not induce c-fos expression with respect to CTRL group (*p* > 0,05). Data are shown as mean ± SEM. Statistical analysis was performed with one-way ANOVA and *posthoc* Tukey’s test (* < 0,05; ** < 0,01; *** < 0,001; **** < *0,00001)* (*n* = 3/group)

To validate if LVC can induce CSD in awake rats, we have measured cortical c-fos expression levels. To eliminate possible surgery- and trepanation-induced c-fos expression in awake animal CSD procedures, we collected CSD-induced cortices from rats under anesthesia for positive controls. These cortices exhibited an approximately fourfold increase in c-fos expression when compared to awake CTRL group (*p* < 0,0001). LVC administration to freely moving awake rats did not induce c-fos expression in any of the cortices (F(2,6) = 103,9; *p* < 0,0001) (Fig. 7).

### Acute LVC or NTG injection do not lead to cortical BBB impairment

**Fig. 8:**
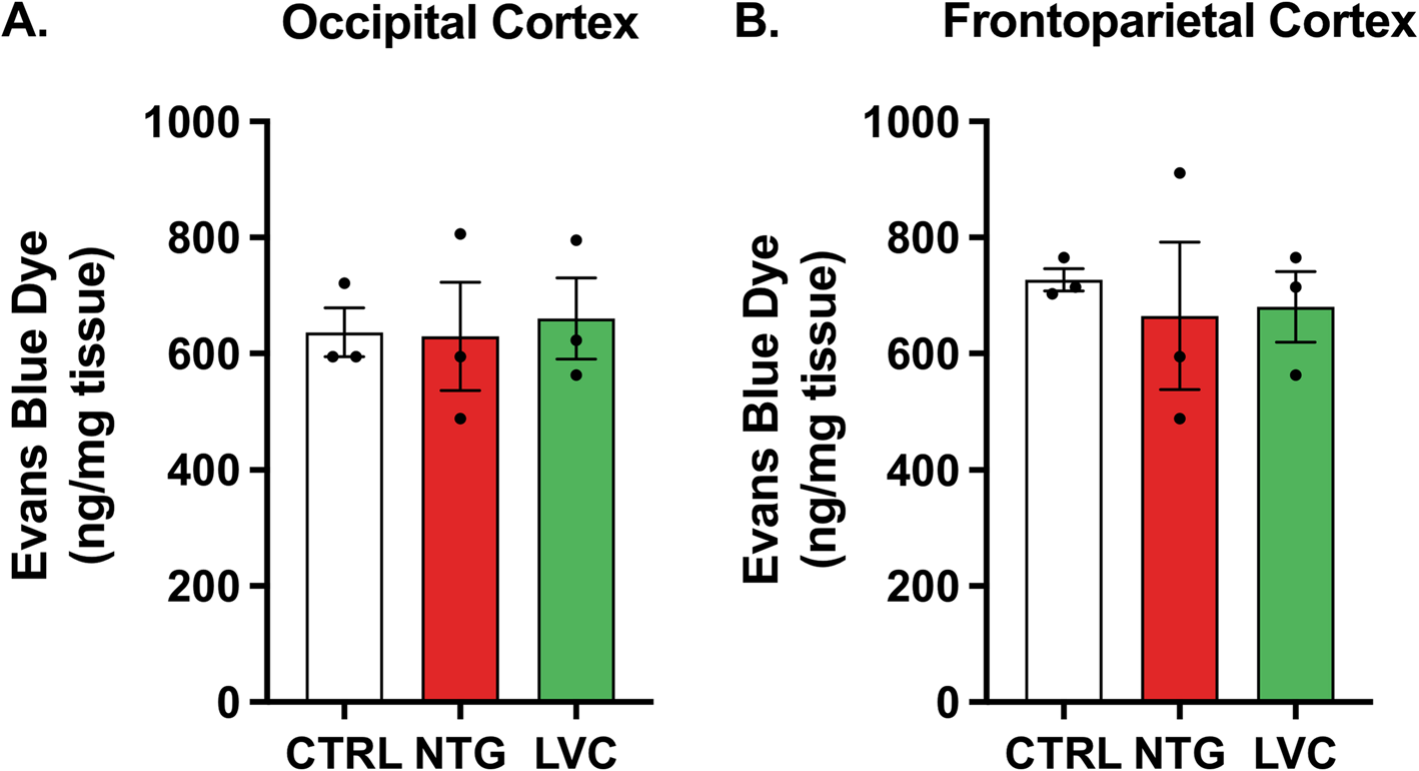
LVC and NTG injection did not disrupt blood–brain barrier. Neither LVC nor NTG led to vascular permeability in occipital and frontoparietal cortex (*p* > 0,05). Data were shown as mean ± SEM. Statistical analysis was performed with one-way ANOVA and *posthoc* Tukey’s test (*n* = 3/group)

To investigate pro-neuroinflammatory effects of acute LVC or NTG injection, we performed EB assay with frontoparietal and occipital cortices. No statistically significant difference was found between EB dye concentrations in the frontoparietal and occipital cortices of LVC- and NTG-injected rats [F(2, 6) = 0,05054; *p* > 0,05 and F(2, 6) = 0,1570; *p* > 0,05, respectively] (Fig. 8A, B).

## Discussion

Our study yields several significant and novel findings: Acute LVC treatment can robustly reduce periorbital and hind paw mechanical thresholds in rats. To our knowledge, LVC, with a 30-min onset, is the fastest migraine-like phenotype-inducing agent in rodents when compared to other systemic migraine-inducing substances, such as well-established NTG, cilostazole and CGRP. In addition, we found LVC-injected rats exhibit other nocifensive behaviors, such as anxiety-like behaviors and photophobia. We also demonstrated a single injection of LVC does not induce CSD in neither anesthetized nor awake freely moving rats, as evident from our dural EEG recordings and lack of increased cortical c-fos expression, respectively. LVC does not affect the electrophysiological parameters of a chemically induced CSD and does not alter SSEP configurations. It is worth noting that acute LVC and NTG injection does not increase cortical BBB permeability.

Mechanical allodynia in the periorbital region is accepted to be the gold standard readout of migraine-like headache in rodents [39]. Periorbital and hind paw withdrawal responses are used to model cephalic and extracephalic hypersensitivity seen in migraineurs [40, 41]. We demonstrated that LVC reduces periorbital and hind paw mechanical thresholds. We detected lowest mechanical thresholds within the first hour of LVC injection, completely recovering at the 2^nd^ hour. This finding is in line with the clinical studies, where LVC administration provoked migraine attack as early as 10 min in some patients [7]. Previous studies with LVC-induced chronic migraine model adopted the 2^nd^ hour as the testing time for LVC post-treatment responses. This may be the reason why lower thresholds were not consistently observed during the establishment of chronic migraine model in these studies [30, 42, 43]. We observed a similar degree of threshold reduction in both LVC and NTG groups, however in the LVC group, decay time of threshold was significantly shorter in comparison to NTG group. Such a rapid reduction and recovery most likely point out to a functional neurobiological change. An increase in the expression of neuroinflammatory genes and newly synthesized proteins cannot be excluded as underlying factors, however their contribution is very unlikely as this would not explain the rapid change in the mechanical sensitivity. In a previous study, intraplantar and intracerebroventricular LVC administration did not change the mechanical sensitivity to von Frey filaments [43]. This finding may suggest the lack of direct central and local mechanism of action and may point out a vascular mechanism. Indeed, all migraine-inducing substances, including LVC, have vasodilatory properties and mechanistically open potassium channels, probably also recruit CGRP signaling [30, 44]. K_ATP_ subunit Kir6.1-knockout mice were protected from NTG- and LVC-induced mechanical allodynia [43]. In addition, LVC-induced mechanical allodynia was reversed by a nonspecific potassium channel blocker, glibenclamide in rodents [45]. On the other hand, glibenclamide failed as an anti-migraine agent in a clinical trial [46]. This discrepancy may be explained by interspecies differences in potassium channel morphology and/or higher doses used in animal experiments [47]. These findings indicate highly selective and subtype-specific potassium channel blockers may provide a novel therapeutic avenue for migraine patients.

As mechanical threshold reduction alone cannot fully explain headache-like behavior in rats, we also assessed anxiety and photophobia, well-known symptoms that commonly accompany migraine attacks [48, 49]. Acute NTG administration is already known to provoke anxiety-like behaviors and light aversion in rats [27, 28, 50]. In our experiments, LVC-injected rats have similarly exhibited anxiety-like behavior and light aversion. Rats naturally tend to avoid light, which complicates studying anxiety-like behavior and hypersensitivity towards light. To rule out possible confounding effects of light on anxiety-like behaviors, we have conducted open field arena test and used time spent in the center zone and number of entries to center zone as measures of light-independent anxiety-like behavior [36]. LVC- and NTG-injected rats spent less time in the center zone and entered the zone fewer times, which confirmed our EPM findings. Our results clearly suggest that LVC can efficiently model migraine-associated anxiety and photophobia, which further validates our model.

Migraine aura is commonly accepted as the clinical correlate of the cortical spreading depolarization phenomenon. Considering LVC can provoke aura in migraineurs with aura, we investigated if LVC could ignite CSD or had any facilitatory effect on KCl-induced CSD. Effects of LVC were compared to NTG, an agent that was found to be incapable of inducing aura in clinical studies. In our studies neither LVC nor NTG induced CSD. On the other hand, NTG increased CSD susceptibility, whereas LVC increased propagation failure rates. Propagation failure can be simply defined as the inability of a CSD wave to spread beyond a certain point. Aura of migraineurs is a phenomenon characterized by migrating symptoms and hypothesized to be the result of a propagating CSD wave. Therefore, reduction in CSD wave propagation may be attributed to the absence of aura with NTG administration clinically. Seemingly controversial to our results, in a previous study, NTG did not alter the CSD threshold [8]. However in this study we conducted our experiment at the peak of mechanical threshold reduction (2^nd^ hour) instead of the first hour as in the aforementioned study. Hence, the difference in the time points can expound the discrepancy between these two studies. Our results exhibited that LVC treatment slightly increased CSD susceptibility without a velocity change. K^+^ channel openers are theoretically proposed to induce and/or facilitate CSD via increased extracellular K^+^ ions [7, 14]. Yet, we have demonstrated that acute LVC yielded a migraine-like phenotype with direct or indirect pro-nociceptive action without inducing and/or facilitating CSD. In brief, headache phenotype provoked by acute LVC is a distinct phenomenon from CSD in our experimental model. The latter was also supported by the result that comparable effects on aura-correlate CSD was observed by both migraine-provoking agents, namely LVC and NTG, although NTG is generally considered as a migraine without aura-provoking agent.

Numerous studies have pointed out that migraine patients have defective sensory gating as demonstrated by somatosensory evoked potentials [51]. This study was the first to examine SSEPs in NTG-, LVC- and CSD-induced migraine models. Our experiments did not reveal significant changes in amplitudes or latencies-to-peak. Evoked potential latencies can be utilized as a tool to assess neuronal hyperexcitability in different rodent and human models [52, 53]. Shorter latencies are thought to signify increased likelihood of faster and simultaneous firing of a neuron population. Neuronal excitability is dependent on resting membrane potential, firing threshold, excitatory input, and inhibitory tonus. As a matter of fact, increased glutamatergic excitation was demonstrated to shorten VEP latencies in a cortical injury model [52]. We have detected a prolongation of latencies in LVC and NTG groups after CSD. CSD is known to cause a shift in the excitatory/inhibitory balance towards inhibition and decrease presynaptic glutamate release probability [54]. We speculate that the increased inhibition and decreased excitation due to CSD may have cause this prolongation of SSEP latencies in NTG- and LVC-treated rats.

Purportedly, migraine aura is associated with altered cortical activity as indicated by fMRI studies [55, 56]. Cortical neuronal activity can be measured with power spectrum density as specific oscillations within certain frequency bands. Vinogradova et al. report power spectrum density alterations in a preclinical migraine with aura model [57]. Our experiments revealed that LVC has a distinct power spectrum density profile from CTRL and NTG groups regarding the post-CSD electrocorticogram. Weaker cortical activity following CSD in LVC-treated animals suggests impaired recovery after CSD induction. This could be explained by potassium channel opener properties of LVC, considering intracellular potassium plays a crucial role in neuronal activity. Increased opening of potassium channels can impede the restoration of ionic imbalances and thus prolong recovery period after CSD in LVC-treated rats.

Absence of expected effects of LVC in electrophysiology experiments may be attributed to the anesthesia as migraine aura is naturally an “awake” phenomenon. We have shown that anesthesia could interfere with the propagation of CSD to subcortical structures such as thalamic reticular nucleus [58–60]. Subsequently, anesthesia may have disturbed the cortex-subcortex relationship and masked possible effects of LVC. CSD is known to generate an enormous amount of c-fos expression in cortex [61]. To exclude the effects of anesthesia, we have injected LVC to awake rats and harvested their cortices after 3 h to allow possible c-fos expression to occur. As our positive control, we have collected CSD-induced cortices from anesthetized animals to avoid possible surgery-induced c-fos expression due to cortical and dural injuries. LVC did not induce c-fos expression in the cortices of awake and freely moving rats. These results showed that acute LVC injection did not induce an CSD activity in awake and freely moving rats.

To investigate whether acute LVC or NTG injection directly disrupts cortical blood–brain barrier and if a possible CSD-induced blood brain barrier disruption accompanies LVC-induced rapid migraine-like phenotype, we performed Evans Blue assay. We did not detect any increased trapping of EB dye in LVC- and NTG-injected rats, pointing out that there is no substantial increase of vascular permeability in the cortex. It can be deduced that LVC-induced acute migraine model does not involve cortical blood–brain barrier disruption. Our data is compatible with the emerging human imaging studies in that blood brain barrier remains intact during acute migraine attacks [62–64].

One of the limitations in this study is the anesthetic use during electrophysiological experiments. As stated above, anesthesia itself alters neuronal excitability and interferes with the communication between different parts of the brain. Thus, it may have masked the effects of our experimental interventions. Lack of female rats is another limitation, since migraine is a disorder with a propensity to affect females more than males. We have used only male rats in our experiments for the sake of comparability with the previous research.

We showed for the first time that systemic LVC 1) induce a fast and narrow window where migraine-like phenotype is manifested in rats, 2) do not ignite CSD within this nociceptive window, 3) do not significantly alter KCl-induced CSD properties except for increased propagation failure, 4) do not significantly modulate somatosensory evoked potentials. Further studies should focus on replicating our study with female rats and using different routes of drug administration to further elaborate on site and mechanism of action. Furthermore, therapeutic validity should also be tested by using established acute migraine attack treatments, such as 5-HT1B/1D agonists, 5-HT1F agonists and/or CGRP antagonists [9, 11, 42]. Besides, clinical trials with larger samples are still needed to further grasp the levcromakalim-induced migraine headache.

In conclusion, acute LVC induces migraine-like phenotype in rats within a comparable timeframe to clinical studies. And this LVC-induced acute migraine model is independent of cortical spreading depolarizations. The fastest manifestation and recovery of migraine-like symptoms in rats by systemic administration of LVC suggests functional alterations are likely induced by K^+^ channel opening properties of LVC in the trigeminal nociceptive system. The detailed research is warranted to understand mechanisms of this narrow window, which may provide novel targets and opportunity to interfere in migraine patients.

## Data Availability

Data will be made available upon reasonable request.

## Abbreviations

LVC: Levcromakalim
NTG: Nitroglycerin
CGRP: Calcitonin gene-related peptide
CSD: Cortical spreading depolarization
BBB: Blood–brain barrier
CTRL: Control
OFA: Open field arena
EPM: Elevated plus maze
DLB: Dark–light box
SSEP: Somatosensory evoked potential
EB: Evans blue
SEM: Standard error of mean
